# Maternal and child health network: multi-partners engagement in improving health planning process in Papua, Indonesia

**DOI:** 10.1186/1471-2458-14-S1-O13

**Published:** 2014-01-29

**Authors:** Digna Niken Purwaningrum

**Affiliations:** 1 Center for Health Policy and Management, Gadjah Mada University, Yogyakarta 55281, Indonesia

## Background

Maternal and child mortality rates in Indonesia have stagnated and even tend to increase for infant mortality, especially in areas with geographical barriers such as eastern Indonesia. Integrated approach in health planning process needs to be established to improve the planning capacity and to maximise potential collaboration between health-related stakeholders. This study aimed to identify the potential role of maternal and child health (MCH) network forum in improving health-planning process at sub-national level.

## Materials and methods

This study used qualitative approach. First assessment of the previous MCH planning process was conducted by in-depth interviews with district health-planning team. We identified the potential MCH-related offices based on the first assessment. Multi-partners workshop was conducted to identify barriers and possible mutual solutions (using bottleneck analysis framework). Final assessment was conducted by evaluating the 2013 planning documents and identifying innovative strategies for maternal and child health programmes resulting from the multi-partners workshop.

## Results

The MCH network forum is a potential mechanism to integrate health-related stakeholders in health planning process. The bottleneck analysis conducted through this forum had identified the main obstacles in the delivery of health services, including: limited number of midwives in remote areas, limited accessibility to health facilities, low quality of data recording and reporting, and lack of health workers able to handle emergency situations. Some of these barriers can be addressed through DHO planning, but other barriers should be addressed through crosscutting funding scheme. Through this forum, health-related stakeholders have managed to include some strategies in their planning documents. For instance, the social service office developed health-related plans including building maternity waiting home to improve health care accessibility for remote population.

## Conclusions

MCH network forum could improve health-planning coordination, health funding utilisation efficiency and ensure that MCH problems are being addressed by wider stakeholders at sub-national level.

